# Expression of Components of the Renin-Angiotensin System in Pyogenic Granuloma

**DOI:** 10.3389/fsurg.2019.00013

**Published:** 2019-04-09

**Authors:** Jessica C. Papali'i-Curtin, Helen D. Brasch, Bede van Schaijik, Jennifer de Jongh, Reginald W. Marsh, Swee T. Tan, Tinte Itinteang

**Affiliations:** ^1^Gillies McIndoe Research Institute, Wellington, New Zealand; ^2^Centre for the Study and Treatment of Vascular Birthmarks, Wellington Regional Plastic, Maxillofacial and Burns Unit, Hutt Hospital, Wellington, New Zealand

**Keywords:** pyogenic granuloma, vascular tumor, renin-angiotensin system, embryonic stem cells, angiotensin converting enzyme

## Abstract

**Background:** There is a growing body of research demonstrating expression of the renin-angiotensin system (RAS) by a putative embryonic stem cell (ESC)-like population within vascular anomalies. This study investigated the expression of components of the RAS in relation to the putative ESC-like population within pyogenic granuloma (PG) that we have recently reported.

**Methods:** PG samples from 14 patients were analyzed for the expression of components of the RAS: pro-renin receptor (PRR), angiotensin converting enzyme (ACE), angiotensin II receptor 1 (ATIIR1) and angiotensin II receptor 2 (ATIIR2), using 3,3-diaminobenzidine (DAB) immunohistochemical (IHC) staining. Immunofluorescence (IF) IHC staining was performed to localize these proteins on four of the PG samples. RT-qPCR was performed on two snap-frozen PG samples. Western blotting (WB) was performed on one snap-frozen PG sample and two PG-derived primary cell lines.

**Results:** DAB IHC staining demonstrated the expression of ACE, PRR, ATIIR1, and ATIIR2 in all 14 PG tissue samples. RT-qPCR analysis confirmed abundant mRNA transcripts for PRR, ACE, AIITR1 and ATIIR2, relative to the housekeeping gene. WB confirmed the presence of PRR, ATIIR1, and ACE in the PG tissue sample, and PRR and ATIIR1, in the PG-derived primary cell lines. IF IHC staining demonstrated the expression of PRR, ACE, ATIIR1 on the primitive population that expressed NANOG and SOX2 on the ERG^+^ endothelium of the microvessels within PG.

**Conclusion:** We have demonstrated the expression of PRR, ACE, and ATIIR1 by the putative the ESC-like population within PG.

## Introduction

Pyogenic granuloma (PG) is a relatively common benign vascular tumor affecting the skin and mucosa. Also known as *lobular capillary hemangioma*, it is comprised of hyperplastic capillary vessels and fibromyxoid stroma with overlying atrophic or ulcerated epidermis (1). There is a slight overall male preponderance (1).

PG arises spontaneously, in previously injured tissues or within vascular malformations (1) and has been associated with certain medications, such as chemotherapeutic agents (2). PG accounts for 0.5% of skin nodules in the pediatric population, most commonly in the first five years of life (1). *Epulis gravidarum* is a sub-type of PG that occurs during pregnancy, and is thought to be induced by hormonal changes (3) and sometimes grows to giant proportions (4).

PG usually presents as a small (<2 cm), red, friable, sessile or pedunculated nodule that bleeds repeatedly. Most PG occur in the head and neck area, followed by the trunk and the limbs and 12% occur on mucosal surfaces (1).

Treatment of PG includes full thickness excision, shave excision, cautery, systemic steroids or topical treatments (e.g., propranolol and imiquimod cream) with full thickness excision having the lowest recurrence rates (1). Shave excision and pulsed dye laser therapy is effective for PG affecting cosmetically sensitive areas (5). Some lesions, especially *epulis gravidarum*, may resolve spontaneously following delivery, although the frequency is unknown (6).

PG has been attributed to disordered angiogenesis although its pathogenesis remains unclear. The capillaries of the PG appear immature (1) and express embryonic stem cell (ESC) markers suggesting a primitive origin of this tumor (7).

ESCs exist in blastocyst of the pre-implantation embryo and possess the capacity to differentiate into all three germ layers–endoderm, ectoderm and mesoderm (8). This pluripotency differentiates ESCs from adult/somatic stem cells and germ cells. NANOG (9), OCT4 (10), SOX2 (11), and signal transducer and activator of transcription (STAT3) (12) are transcription factors that maintain pluripotency and are markers that characterize ESCs. Once activated, the STAT family of proteins bind target sites on the DNA, of which STAT3 activation is essential for cell self-renewal (13). OCT4 is a POU family transcription factor that interacts with SOX2 to maintain ESC pluripotency and is expressed on early ESCs (10). SOX2 is a transcription factor from the sex-determining Y family which is critical in neural cells and ESCs and also important in multiple stages of mammalian development (11). SOX2 and OCT4 act synergistically to regulate NANOG, a homeobox-containing transcription factor essential in maintaining pluripotency of the inner cell mass (9).

We have reported two putative ESC-like subpopulations within PG, one on the endothelium that expresses OCT4, NANOG, pSTAT3 and SOX2, and an interstitial subpopulation that expresses NANOG, pSTAT3 and SOX2 (7). We infer that the ESC-like population on the endothelium may differentiate to form the downstream interstitial subpopulation resulting in the loss of OCT4 expression, or alternatively, de-differentiation of the interstitial cells may give rise to the more primitive endothelial subpopulation.

Expression of components of the RAS in venous malformation (VM) (14) and infantile hemangioma (IH) (15) has been reported. This observation underscores the efficacy of RAS modulators in the treatment of IH using β-blockers(16) and angiotensin converting enzyme (ACE) inhibitors (15, 17, 18). In addition to the classical role in cardiovascular homeostasis, the role of the RAS in cellular proliferation, angiogenesis, immune response and apoptosis is increasingly appreciated (19, 20). Components of the RAS are present physiologically in the vasculature, liver, pancreas, kidney, brain and reproductive organs, and they have also been demonstrated in multiple types of malignant and benign tumors, often in different concentration than the normal surrounding tissues (21–23).

The key components of the RAS include pro-renin receptor (PRR), which converts pro-renin to active renin (24); ACE which converts inactive angiotensin I (AT1) to active angiotensin II (ATII) (24); angiotensin II receptor 1 (ATIIR1) which binds with ATII and couples with signaling molecules to cause multiple effects such as vasoconstriction and cellular proliferation (25); and angiotensin II receptor 2 (ATIIR2) which functions similarly to ATIIR1 but appears to cause the opposite downstream signaling effects such as vasodilation and inhibition of cell growth (23). PRR has been implicated in many types of cancer and is thought to play a role in cellular proliferation and apoptosis (26–28). Recent investigations into the RAS in IH demonstrate the presence of ACE and ATIIR2 on the hemogenic endothelium of this tumor, and the proliferative effect of ATII via ATIIR2 on IH-derived cells (15, 17). ATIIR1 appears to play a lesser role in IH (17). The expression of the ACE and ATIIR2 on the hemogenic endothelium in IH underscores the accelerated involution of proliferating IH induced by β-blockers (16) and ACE inhibitors (18).

In this study we investigated the expression on the aforementioned components of the RAS within PG at both the transcriptional and translational levels, and their localization in relation to the putative ESC-like population.

## Materials and Methods

### Tissue Samples

PG tissue samples from four female and 10 male patients with an average age of 21.3 (range, 3–42) years were sourced from the Gillies McIndoe Research Institute Tissue Bank and used for this study which was approved by the Central Regional Health and Disability Ethics Committee (Ref. 13/CEN/130). Written consent was obtained from participants or their caregivers in accordance with the Declaration of Helenski. The diagnosis of PG was made clinically including a history of an acquired lesion with the typical histopathological appearance and the absence of GLUT-1 staining (29). The lesions were located in the head and neck area (*n* = 8) and the hand (*n* = 6), measuring 8–25 (mean, 12) mm, and were present for 2–10 (mean, 3) months prior to excision.

### 3,3-Diaminobenzidine Immunohistochemical Staining

3,3-Diaminobenzidine (DAB) immunohistochemical (IHC) staining was performed on 4 μm-thick formalin-fixed paraffin-embedded sections of 14 PG tissue samples using the Leica Bond Rx auto-stainer (Leica, Nussloch, Germany) with antibodies against PRR (1:100; cat# HPA003156, Sigma-Aldrich, St. Louis, MO, USA), ACE (1:40; cat# 3C5, Serotec, Raleigh, NC, USA), ATIIR1 (1:25; cat# ab9391, Abcam, Cambridge, MA, USA), ATIIR2 (1:2000; cat# NBPI-77368, Novus Biologicals, Littleton, CO, USA), SOX2 (1:200; cat# PA1-094, Thermo Fisher Scientific, Rockford, IL, USA), NANOG (1:100; cat# ab80892, Abcam) and ERG, (1: 200; Cell Marque, Rocklin, CA, USA). Antibodies were diluted with bond primary diluent (Leica). DAB IHC-stained slides were mounted in Surgipath Micromount (cat# 3801732, Leica).

Immunofluorescence (IF) IHC staining was performed on four PG tissue samples of the original cohort of 14 patients used for DAB IHC staining, using the same antibodies dilutions and the following combinations NANOG/ACE, SOX2/ACE, PRR/ACE, ERG/ATIIR1. For IF IHC detection, a combination of Vectafluor Excel anti-rabbit 594 (ready-to-use; cat# VEDK-1594, Vector Laboratories, Burlingame, CA, USA) and Alexa Fluor anti-mouse 488 (1:500; cat# A21202, Life Technologies, San Diego, CA, USA) were used to detect combinations that included PRR and ATIIR2, and Vectafluor Excel anti-mouse (ready-to-use; cat# VEDK2488, Vector Laboratories) and Alexa Fluor anti-rabbit 594 (1:500; cat# A21207, Life Technologies) to detect combinations that included ACE and ATIIR1. IF IHC-stained slides were mounted in Vectashield HardSet antifade mounting medium with DAPI (cat# H-1500, Vector Laboratories).

The human tissue samples used for positive controls were placenta for PRR (30), kidney for ACE (31), and ATIIR2 (32), and liver for ATIIR1 (32), as previously reported. Negative controls were performed by staining PG tissue samples by omitting the primary antibodies, to determine their specificity.

### PG-Derived Primary Cell Lines

Primary cell lines were derived from two fresh surgically excised PG tissue samples by culturing them as explants in Matrigel and then extracting the cells following abundant growth, as previously described (15). The extracted PG cells were then cultured and passaged in DMEM medium (cat# 10569010, Gibco, Thermo Fisher Scientific, Waltham, MA, USA) supplemented with 1% FCS (cat# 10091148, Gibco, Thermo Fisher Scientific), 5% mTeSR™ (cat# 85850, StemCell Technologies, Vancouver, Canada), 1% penicillin-streptomycin (cat# 15140122, Gibco, Thermo Fisher Scientific) and 0.2% gentamicin/amphotericin B (cat# R01510, Gibco, Thermo Fisher Scientific). All cultures were maintained in a humidified incubator at 37°C at an atmosphere of 94% air and 5% CO_2_. All primary cell lines used for the experiments were passages 6–8.

### NanoString mRNA Analysis

NanoString mRNA analysis was performed on six snap-frozen PG tissue samples of the original cohort of 14 patients used for DAB IHC staining, using the NanoString nCounter™ Gene Expression Assay (NanoString Technologies, Seattle, WA, USA). RNA was extracted from the samples using RNeasy Mini Kit (Qiagen, Hilden, Germany) and subjected to the NanoString nCounter™ Gene expression assay performed by New Zealand Genomics Ltd (Dunedin, New Zealand) according to the manufacturer's protocol. Probes for the genes encoding ACE (NM_000789.2), PRR (ATP6AP2, NM_005765.2), ATIIR1 (NM_000685.3) and ATIIR2 (NM_000686.3), and housekeeping gene GAPDH (NM_002046.3) were designed and synthesized by NanoString Technologies. The raw data were analyzed by nSolverTM software (NanoString Technologies) using standard settings and normalized against the housekeeping gene.

### RT-qPCR

To confirm transcription activation of components of the RAS in PG-derived primary cell lines from two PG tissue samples of the original cohort of 14 patients included for DAB IHC staining. RNA samples were then prepared using the RNeasy Mini Kit (Qiagen), with RNA extracted through DNase digest using the QIAcube (Qiagen). Samples were then subjected to NanoDrop 2000 Spectrophotometer (Thermo Fisher Scientific) quantification. Extractions for each sample were performed in triplicates and analyzed. RNA was analyzed with Rotor-Gene Multiplex RT-PCR Kit (Qiagen) and subjected to RT-qPCR using the Rotor-Gene Q (Qiagen). The expression of ESC markers was detected using gene-specific TaqMan (Thermo Fisher Scientific cat# 4331182) primers-probes (AT2R1: Hs00258938_m1, AT2R2: Hs02621316_s1, ACE: Hs00174179_m1, ATP6AP2/PRR: Hs00997145_m1), and the reference gene GAPDH (Hs99999905_m1). Positive controls were demonstrated on uterine fibroid tissue, and specificity of probes were confirmed by inclusion of a water negative control.

### Western Blotting

Western blotting (WB) was performed on total protein extracts from one snap-frozen PG tissue section and two PG-derived primary cell lines. The protein extracts were resolved by 4–12% one-dimensional polyacrylamide gel electrophoresis then transferred to polyvinylidene difluoride membranes. The samples were them probed using the primary antibodies ACE (1:200; cat# sc-12184, Santa Cruz, Rockford, IL, USA), PRR (1:500; cat# ab40790, Abcam, Cambridge, UK), ATIIR1 (1:200; cat# sc-1173, Santa Cruz), ATIIR2 (1:500; cat# ab92445, Abcam) and β-actin (1:500; cat# ab8229 Abcam); then incubated with the appropriate secondary antibody: goat anti-rabbit HRP (1:1000, cat# A16110, Thermo Fisher) and donkey anti-goat HRP (1:1,000, cat# ab97120, Abcam). ACE was probed using a tertiary cascade consisting of a rabbit anti-goat Superclonal™ biotin conjugated secondary antibody (1:4,000; cat# A27013, Thermo Fisher) followed by a Pierce™ Streptavidin Poly HRP (1:5000, cat# 21140, Thermo Fisher) at 4°C for 10 min. Clarity Western ECL (cat# 1705061, Bio-Rad) was used as the substrate for visualizing HRP detected protein bands and the ChemiDoc MP Imaging System (Bio-Rad) and Image Lab 5.0 software (Bio-Rad) were used for band detection and analysis. Positive controls were human placenta for PRR and ATIIR1, mouse lung for ACE, and a recombinant ATIIR2 protein (cat# H00000186-P01, Novus Biologicals, Littleton, CO, USA) for ATIIR2. Matched mouse (1:500; cat# ab18443, Abcam) and rabbit (1:500; cat# ab171870, Abcam) isotype controls were used as appropriate negative controls.

### Image Capture and Analysis

All DAB IHC-stained slides were viewed and photographed using the Olympus BX53 bright field microscope with an Olympus DP21 digital camera (Olympus, Tokyo, Japan). The Olympus FV1200 confocal microscope (Tokyo, Japan) was used for the IF IHC-stained slides with subsequent 2D deconvolution using CellSens Dimension 1.11 software (Olympus).

### Statistical Analysis

Statistical analysis to determine significant differences between the NanoString mRNA results were analyzed using paired *t*-tests of the SPSS (v22, IBM).

## Results

### Histology

PG was comprised of microvessels organized into lobules embedded in fibromyxoid stroma (Supplementary Figure 1A).

### DAB Immunohistochemical Staining

**Figure 1:**
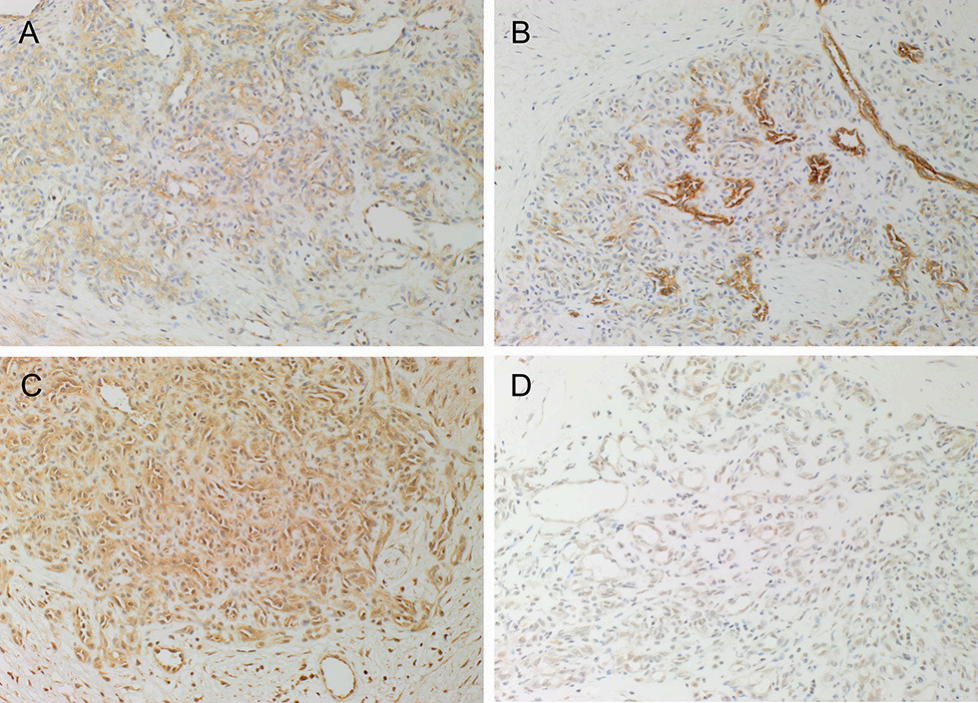
Representative 3,3-diaminobenzidine immunohistochemical-stained sections of pyogenic granuloma demonstrating the expression of PRR (**A**, brown) on the endothelium of the microvessels and cells within the perivascular tissue. ACE (**B**, brown) was expressed on the endothelium of the microvessels. ATIIR1 (**C**, brown) was expressed on the endothelium of the microvessels and cells within the perivascular tissue. Weak staining of ATIIR2 (**D**, brown) on the endothelium of the microvessels and cells within the perivascular tissue was observed. Nuclei were counterstained with hematoxylin (blue). Original magnification 200x.

DAB IHC staining demonstrated the expression of PRR (Figure 1A, brown) on the endothelium of the microvessels and cells within the perivascular tissues, while ACE (Figure 1B, brown) was expressed on the endothelium of the microvessels, in PG. ATIIR1 (Figure 1C, brown) staining was strongest on the endothelium of the microvessels and cells within the perivascular tissue. ATIIR2 (Figure 1D, brown) was also expressed on the endothelium of the microvessels in PG.

Expected staining patterns for PRR (Supplementary Figure 1B, brown), ACE (Supplementary Figure 1C, brown), ATIIR1 (Supplementary Figure 1D, brown), and ATIIR2 (Supplementary Figure 1E, brown) were demonstrated on human placenta, kidney, liver, and kidney, respectively. Staining with the isotype controls in a PG sample provided an appropriate negative control (Supplementary Figure 1F).

### Immunofluorescence Immunohistochemical Staining

**Figure 2:**
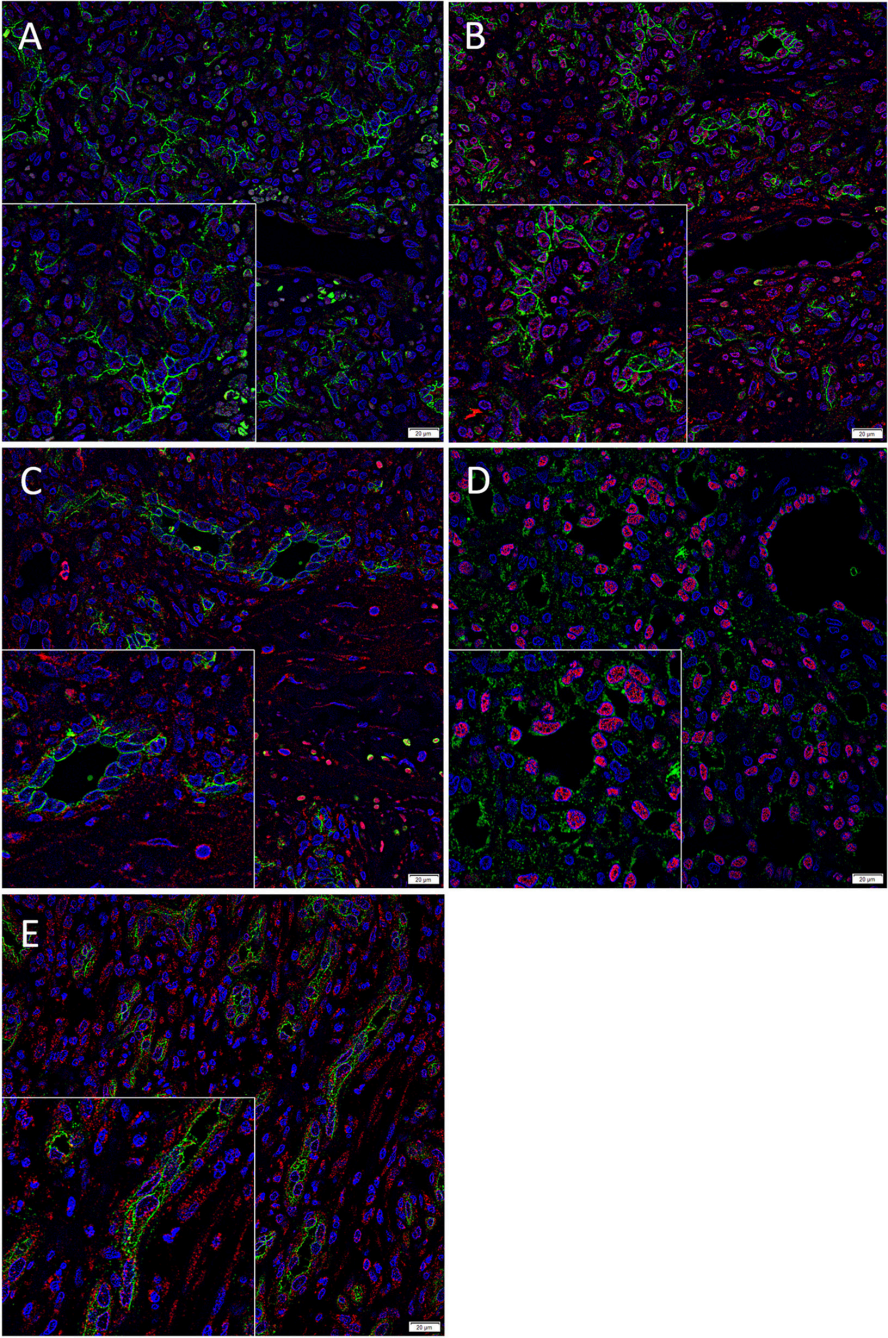
Representative immunofluorescence immunohistochemical-stained sections of pyogenic granuloma demonstrating the expression of ACE (**A,B**, green) on the SOX2^+^ (**B**, red) endothelium of the microvessels but not the NANOG^+^ (**A**, red) cells within the perivascular tissue. The ACE^+^ (**C**, green) endothelium of the microvessels and the perivascular cells within PG expressed PRR (**C**, red). The ERG^+^ (**D**, red) endothelium of the microvessels expressed ATIIR1 (**D**, green). PRR (**E**, red) was expressed on both the CD34^+^ (**E**, green) endothelium as well as the non-endothelial cells. Nuclei were counterstained with 4′,6′-diamino-2-phenylindole (**A–D**, blue). Scale bars: 20 μm. Inserts demonstrate magnified areas of the image within each figure (magnification 400X).

We have previously demonstrated the presence of two putative ESC-like subpopulations within PG with one subpopulation localized to the endothelium that expressed NANOG (Figure 2A, red) (7). Interestingly only ACE (Figure 2B, green) was expressed by the SOX2^+^ (Figure 2B, red) endothelium of the microvessels but not the NANOG^+^ (Figure 2A, red) cells within the perivascular tissue. The ACE^+^ (Figure 2C, green) endothelium of the microvessels and the cells within the perivascular tissue of PG expressed PRR (Figure 2C, red). The ERG^+^ (Figure 2D, red) endothelium of the microvessels expressed ATIIR1 (Figure 2D, green). ATIIR2 (Figure 2E, red) was expressed on both the CD34^+^ (Figure 2E, green) endothelium of the microvessels and the cells within the perivascular tissue. An insert within each image provides a magnified area to better demonstrate the staining pattern. Split images of IF IHC staining presented in Figure 2 are shown in Supplementary Figure 2. The negative controls demonstrated minimal staining (Supplementary Figure 2K).

### NanoString mRNA Analysis

**Figure 3:**
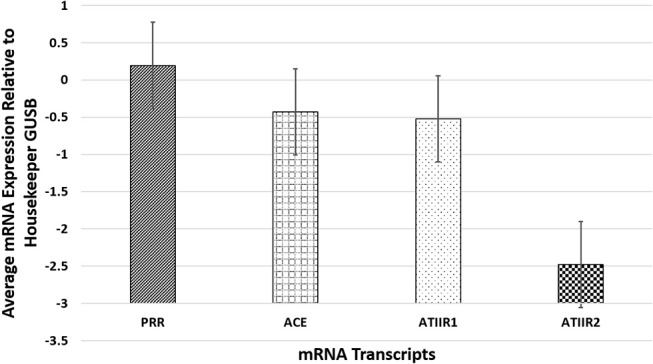
NanoString mRNA analysis confirmed transcriptional activation of PRR, ACE, and ATIIR1 in all six pyogenic granuloma tissue samples examined while ATIIR2 was detected in only one sample, relative to the housekeeping gene GUSB. There were significantly higher amounts of PRR compared to ATIIR1 and ACE (*p* < *0.05*).

NanoString mRNA analysis confirmed transcriptional activation of PRR, ACE, and ATIIR1 in all six PG tissue samples examined but ATIIR2 was detected in only one sample, relative to the housekeeping gene GUSB (Figure 3). Statistical analysis revealed significantly higher amounts of PRR compared to ATIIR1 and ACE (*p* < *0.05*).

### RT-qPCR

**Figure 4:**
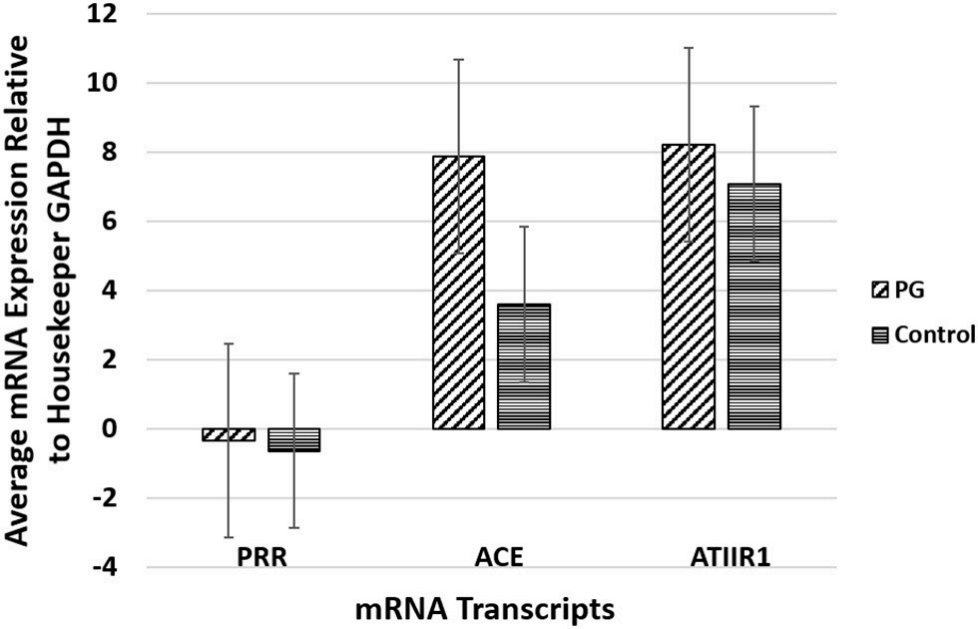
Graph of RT-qPCR performed on two pyogenic granuloma-derived primary cell lines, demonstrating average mRNA expression of PRR, ACE and ATIIR1, relative to housekeeping gene GAPDH, and in comparison to fibroid tissues as a positive control. ATIIR2 mRNA was below the detectable levels.

RT-qPCR performed on the two PG-derived primary cell lines confirmed abundant mRNA expression of PRR, ACE and ATIIR1, while ATIIR2 mRNA was below the detectable levels (Figure 4), relative to the housekeeping gene GAPDH, and in comparison, with uterine fibroid tissues as a positive control.

### Western Blotting

**Figure 5:**
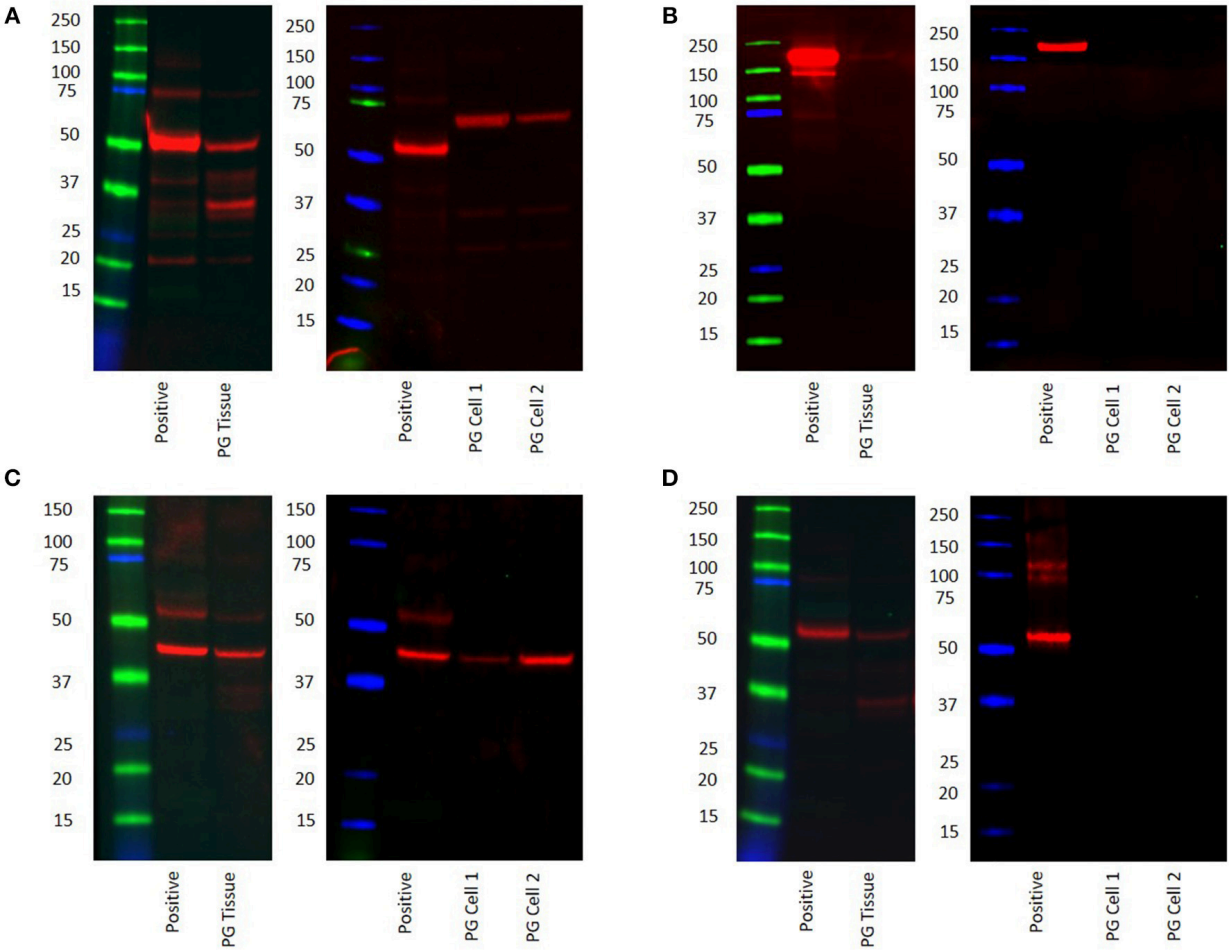
Western blot images of total protein extracted from a snap-frozen pyogenic granuloma tissue sample and two pyogenic granuloma-derived primary cell lines, and the positive control demonstrating the presence of PRR **(A)**, the absence of ACE **(B)**, and the presence of both ATIIR1 **(C)**, and ATIIR2 **(D)**. Each blot ladder is annotated with the molecular size (kDa).

WB confirmed the presence of PRR at the molecular weight of 42 kDa in the PG tissue sample and the two PG-derived primary cell lines at 70 kDa (Figure 5A, red) representing dimerization of PRR (30). ACE was not detected at the expected molecular weight of 194 kDa (22) in both the tissue sample and the primary cell lines (Figure 5B, red). ATIIR1 was detected in both the tissue samples and primary cell lines (Figure 5C, red) with bands at the expected molecular weight of 42 kDa(28). ATIIR2 was below detectable levels in both the tissue and the cell lines (Figure 5D). Bands for β-actin (Supplementary Figure 3A, red) confirmed approximate equivalent protein loading for all tissue samples examined. The respective positive controls were used confirmed specificity of the antibody for their target proteins. The rabbit and mouse IgG isotype controls (Supplementary Figure 3B) were used to detect any non-specific staining and therefore confirmed the presence of the components of the RAS.

## Discussion

This study demonstrated the presence of PRR, ACE, ATIIR1, and ATIIR2 within PG. Interestingly the expression of ATIIR2 was only demonstrated by IHC staining, and this may be potentially due to sampling bias in NanoString and WB analyses. IF IHC staining demonstrated the expression of all four components of the RAS on the endothelium of the microvessels that expressed ESC markers SOX2 and NANOG (7). The putative ESC-like subpopulation within the perivascular tissue that expresses NANOG (7), did not express ACE. Cells within the stroma expressed both PRR and ATIIR2. However, due to the limitation by the antibody sources we were not able to determine whether these were the same cells that express NANOG.

The RAS is classically known as an endocrine system, however, the presence of a paracrine and an intracrine element has been demonstrated (19, 24). Components of the RAS have also been localized to multiple organs, suggesting a local paracrine RAS functioning independently, or in conjunction with, the endocrine RAS (20). Our study supports the presence of a local RAS within PG. Angiotensinogen (AGN) is a protein physiologically secreted by the liver into the general circulation. Pro-renin is physiologically converted to renin by the juxtaglomerular cells of the kidney; renin then cleaves AGN to form the decapeptide ATI. ATI is primarily converted to ATII by ACE by removing the histidyl-leucine dipeptide from the carboxyl end of AT1 to create an octapeptide (33, 34). The presence of ACE on the endothelium of the microvessels in the PG samples suggests paracrine conversion of ATI to active ATII and the downstream effects of ATII may play a role in cellular proliferation and/or angiogenesis of the PG (23, 24, 35).

PRR binds pro-renin which then undergoes conformational change (24) creating a four-fold increase in the catalytic conversion of AGN to ATI (30). The presence of PRR on the endothelium of the microvessels in PG suggests a local effect downstream of ATI.

The localization of ATIIR1 to the endothelium of the microvessels of PG suggests that ATIIR1 may contribute to the formation of immature microvessels in PG, possibly through preferential differentiation down an endothelial phenotype (36).

ATIIR2 was detected by IHC staining in our study with a weak and diffuse staining pattern, but was not detected by NanoString, RT-qPCR, and WB analyses. Interestingly ATIIR2 contributes to angiogenesis of the endothelial stem cells via upregulation of VEGF and increasing expression of VEGF2 receptors (35). This is in contrast to our finding in IH that ATIIR2 agonist facilitated cellular proliferation (17). This may be because ATIIR2 has been primarily located in fetal tissue (37) and that IH is a tumor of infancy, possibly at a time when this receptor is more abundant (37).

In light of reports of the presence of hemangioblast stem cell population on primitive-phenotypic vessels (36), it is exciting to speculate that PG consist of hemangioblast-like cells with a predominant endothelial differentiation pathway. This may be predominantly through ATIIR1 signaling, however, this requires further investigation.

Our study adds to the growing body of evidence of the involvement of the RAS in vascular anomalies including VM, IH and PG. To the best of our knowledge, this is the first study demonstrating the presence and localization of PRR, ACE, and ATIIR1 to the putative ESC-like population within PG. This novel finding offers insights into the biology of PG with the potential of using RAS modulators such as β-blockers and ACE-inhibitors, for this common and often troublesome tumor.

Limitations of this study include a relatively small sample size, the lack of normal control tissues such as skin for the experiments and functional data on the role of the RAS in PG. Further studies including a larger sample size, inclusion of control tissues such as normal skin, and *in vitro* experiments with sorting of the putative ESC-like cells from PG lesions are needed to determine their expression of the aforementioned components of the RAS, and their response to administration of the RAS peptides.

## Author Contributions

TI and ST formulated the study hypothesis and designed the study. JP-C, HB, TI, and ST interpreted the IHC data. TI and ST interpreted the NanoString data. JdJ performed the RT-qPCR experiments and JdJ, JP-C, TI, and ST interpreted the data. BvS performed the WB experiments. BvS, TI, and ST interpreted the data. RM performed the statistical analysis. JP-C, TI, and ST drafted the manuscript. All authors approved the manuscript.

### Conflict of Interest Statement

TI and ST are inventors of a provisional patent Treatment of Vascular Anomalies (PCT/NZ2017/050032). The remaining authors declare that the research was conducted in the absence of any commercial or financial relationships that could be construed as a potential conflict of interest.

## Supplementary Material

Supplementary Figure 1 A hematoxylin and eosin section of pyogenic granuloma (PG) demonstrating microvessels organized into lobules embedded in a fibromyxoid stroma **(A)**. DAB IHC staining of positive controls for PRR (**B**, brown) on human placenta, ACE (**C**, brown) on human kidney, ATIIR1 (**D**, brown) on human liver, and ATIIR2 (**E**, brown) on human kidney. The negative control on a PG sample using an IgG isotype **(F)** demonstrated no staining. Nuclei were counter-stained with hematoxylin (**A–F**, blue]. Original magnification: 200x.

Supplementary Figure 2 Split images of immunofluorescence immunohistochemical-stained sections of pyogenic granuloma (PG) shown in Figure 2 demonstrating expression of SOX2 (**A**, red) and ACE (**B**, green), NANOG (**C**, red) and ACE (**D**, green), PRR (**E** red) and ACE (**F**, green), ERG (**G**, red) and ATIIR1 (**H**, green), ATIIR2 (**I**, red) and CD34 (**J**, green). A negative control **(K)** to test the specificity of the fluorescent secondary antibodies was performed on a section of PG. Cell nuclei were counter-stained with 4′,6-diamidino-2-phenylindole (**A–K**, blue). Scale bars: 20 μm.

Supplementary Figure 3 Western blot images of a pyogenic granuloma (PG) tissue sample and PG-derived primary cell lines probed for β-actin **(A)** and mouse and rabbit isotype negative control antibodies **(B)**. The blot ladder is annotated with the molecular size (kDa).
