# Supplementary figures and images for: Expression of Components of the Renin-Angiotensin System in Pyogenic Granuloma
Source: Front Surg. 2019 Apr 9;6:13. doi: 10.3389/fsurg.2019.00013 (PMC6465765; doi:10.3389/fsurg.2019.00013)

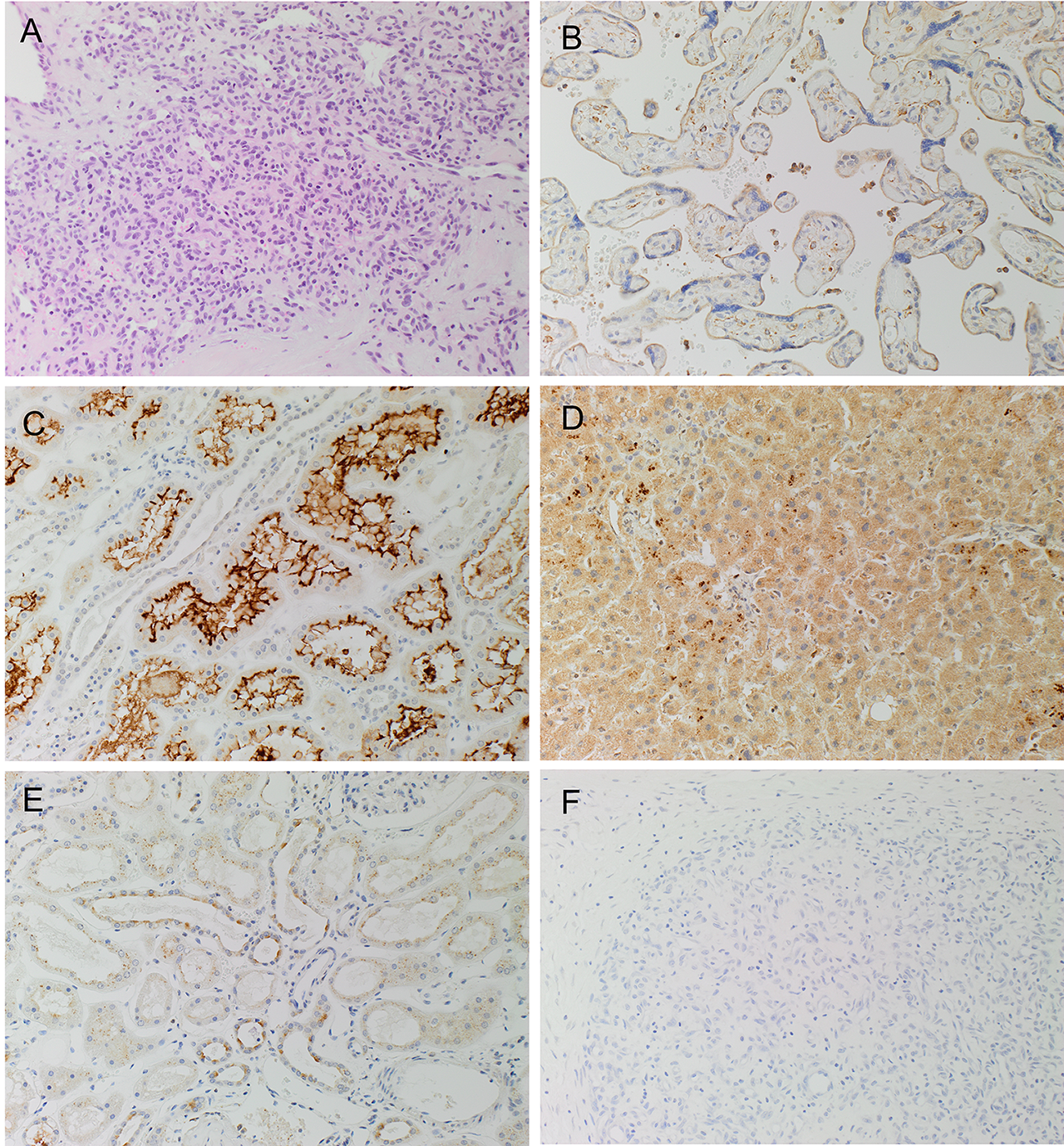

Supplement: Supplementary Figure 1 — A hematoxylin and eosin section of pyogenic granuloma (PG) demonstrating microvessels organized into lobules embedded in a fibromyxoid stroma (A). DAB IHC staining of positive controls for PRR (B, brown) on human placenta, ACE (C, brown) on human kidney, ATIIR1 (D, brown) on human liver, and ATIIR2 (E, brown) on human kidney. The negative control on a PG sample using an IgG isotype (F) demonstrated no staining. Nuclei were counter-stained with hematoxylin (A–F, blue]. Original magnification: 200x. [file Image_1.TIF]

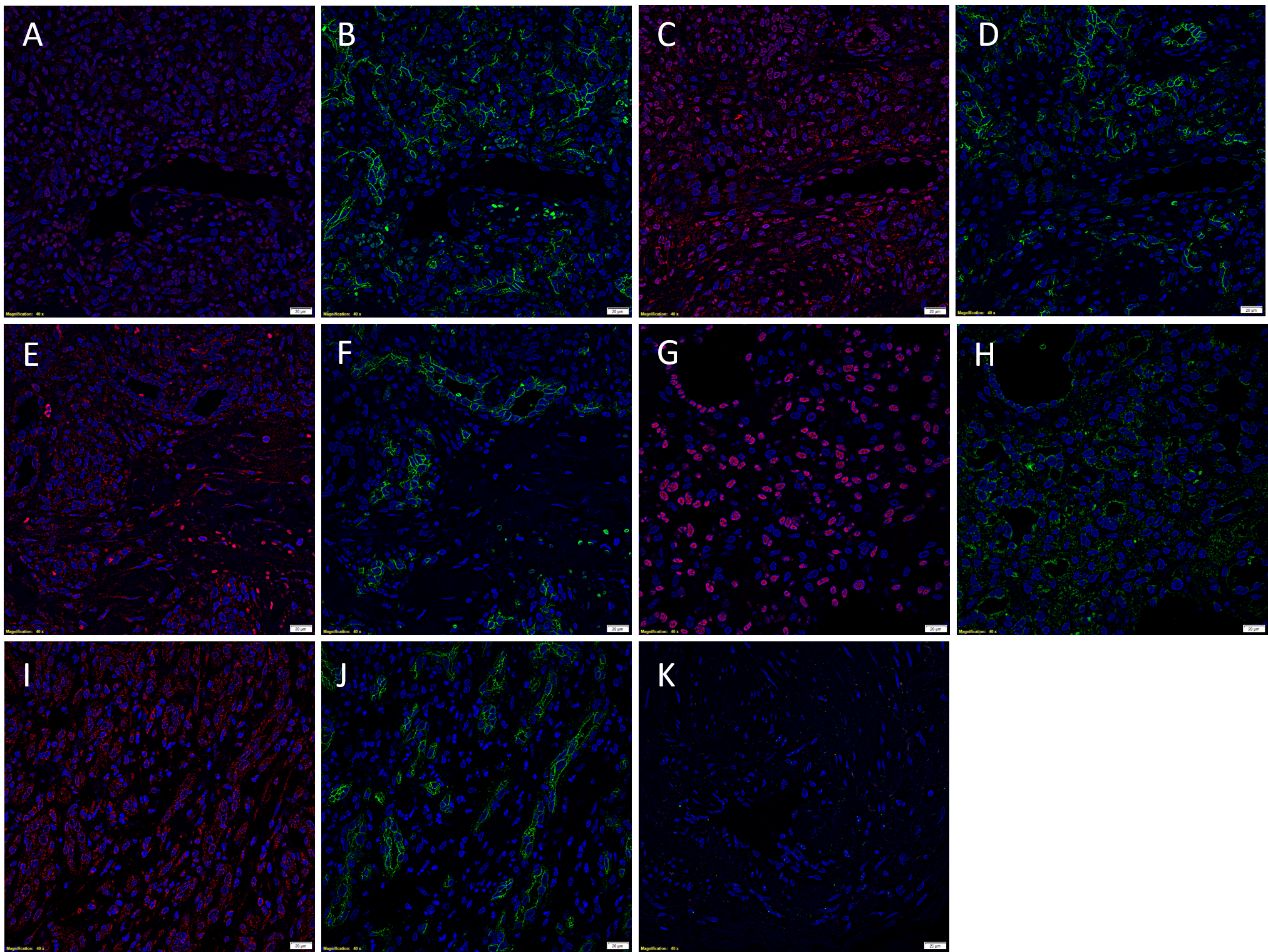

Supplement: Supplementary Figure 2 — Split images of immunofluorescence immunohistochemical-stained sections of pyogenic granuloma (PG) shown in Figure 2 demonstrating expression of SOX2 (A, red) and ACE (B, green), NANOG (C, red) and ACE (D, green), PRR (E red) and ACE (F, green), ERG (G, red) and ATIIR1 (H, green), ATIIR2 (I, red) and CD34 (J, green). A negative control (K) to test the specificity of the fluorescent secondary antibodies was performed on a section of PG. Cell nuclei were counter-stained with 4′,6-diamidino-2-phenylindole (A–K, blue). Scale bars: 20 μm. [file Image_2.TIF]

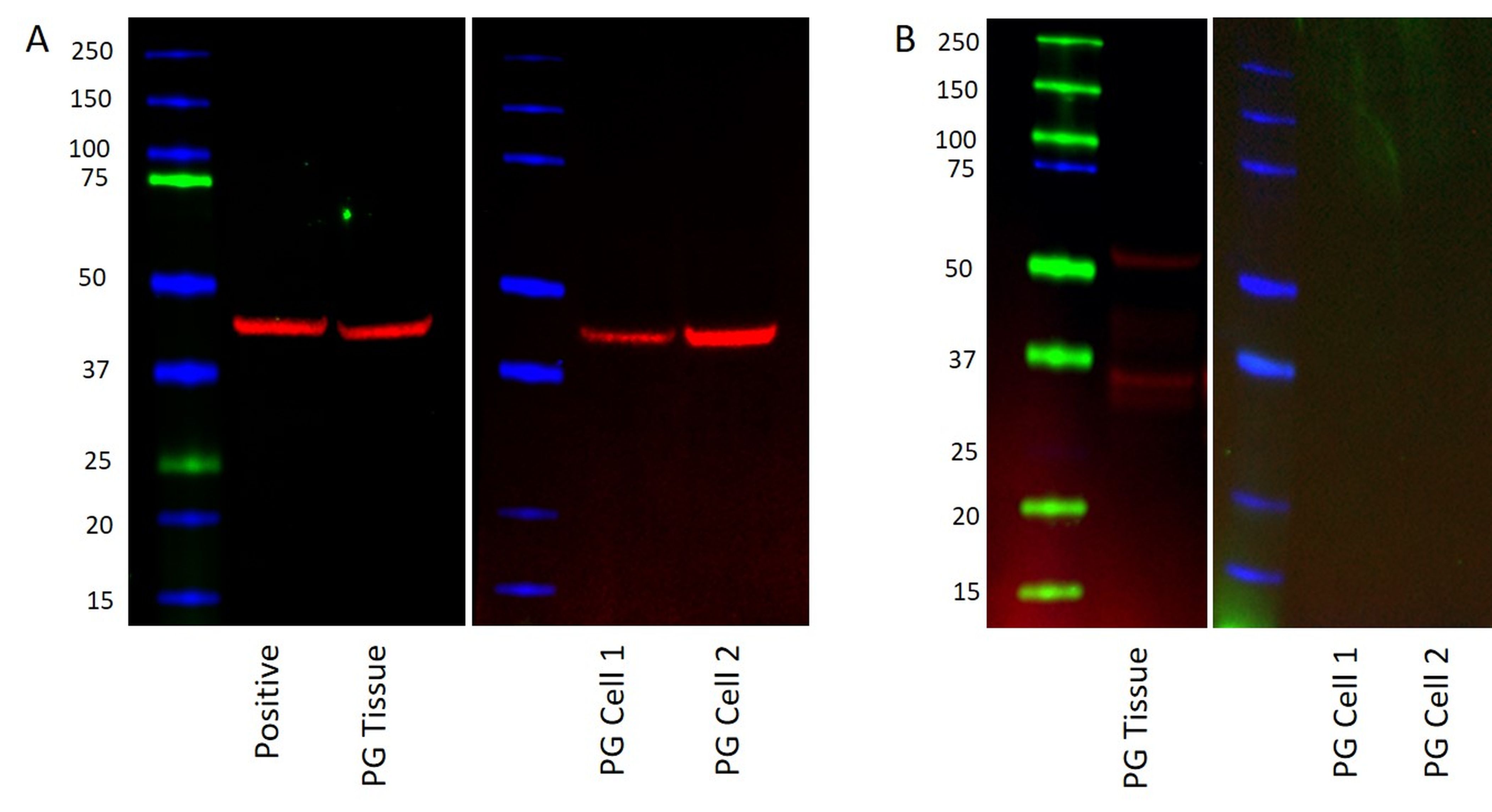

Supplement: Supplementary Figure 3 — Western blot images of a pyogenic granuloma (PG) tissue sample and PG-derived primary cell lines probed for β-actin (A) and mouse and rabbit isotype negative control antibodies (B). The blot ladder is annotated with the molecular size (kDa). [file Image_3.jpg]
